# Molecular Characterization and Functional Effect on Canine Peripheral Blood Mononuclear Cells of an Uncharacterized Major Egg Antigen EGR-01664 from *Echinococcus granulosus*

**DOI:** 10.3390/genes16111384

**Published:** 2025-11-14

**Authors:** Juncheng Huang, Xinwen Bo, Xuke Chen, Jiaxin Zhao, Jianan Zhao, Linying Wei, Yanyan Zhang, Yan Sun, Zhengrong Wang

**Affiliations:** 1 Institute of Animal Husbandry and Veterinary Medicine, Xinjiang Academy of Agricultural and Reclamation Science, Shihezi 832000, China; hjcll20001020@163.com (J.H.); chenxuke1@outlook.com (X.C.); zhaojn0215@163.com (J.Z.); 15241866800@163.com (L.W.); syy1028@126.com (Y.S.); 2 State Key Laboratory of Sheep Genetic Improvement and Healthy Production, Xinjiang Academy of Agricultural and Reclamation Science, Shihezi 832000, China; 3 College of Animal Science and Technology, Tarim University, Alaer 843300, China; 4 College of Animal Science and Technology, Shihezi University, Shihezi 832000, China

**Keywords:** *Echinococcus granulosus*, EGR-01664 gene, bioinformatics, prokaryotic expression, canine PBMCs

## Abstract

Background: Cystic echinococcosis (CE) is a globally distributed zoonosis triggered by the larval stage of *Echinococcus granulosus* (*E. granulosus*), impacting humans and an extensive array of mammalian intermediate hosts. EGR-01664 is the major egg antigen of *E. granulosus*, but almost nothing is currently known about the function of EGR-01664 from *E. granulosus*. Methods: This study aimed to investigate the *E. granulosus* EGR-01664 gene (GenBank ID: 36337379), and the recombinant EGR-01664 protein was expressed successfully. Next, the transcription of the EGR-01664 gene across various developmental stages of *E. granulosus* was analyzed. Its spatial expression patterns in adult worms and protoscoleces were characterized using both quantitative PCR (qPCR) and immunofluorescence assays. Furthermore, the immunomodulatory effects of rEGR-01664 on cell proliferation, nitric oxide production, and cytokine secretion were examined by co-culturing the recombinant protein with canine PBMCs. Results: The rEGR-01664 could be recognized by sera from dogs infected with *E. granulosus*. Immunofluorescence assay (IFA) localization revealed the protein’s presence in the epidermis of protoscoleces, the adult epidermis, and some parenchymal tissues. qPCR revealed that EGR-01664 mRNA levels were significantly higher in protoscoleces compared to adults (*p* < 0.0001). At a concentration of 20 μg/mL, rEGR-01664 could significantly activate the transcription and expression of IL-10, TGF-β1, IL-17A, and Bax in canine PBMCs. However, with an increase in concentration, it inhibited the expression of IFN-γ, Bcl-2, GSDMD, IL-18, and IL-1β. These results suggest that the EGR-01664 gene plays a crucial role in the development, parasitism, and reproduction of *E. granulosus.* In vitro studies have shown that rEGR-01664 protein regulates the immune regulation function of canine PBMCs, suggesting its potential as a vaccine adjuvant or immunotherapy target. Conclusions: EGR-01664 may modulate canine PBMC functions to regulate host immune responses, thereby facilitating our understanding of how *E. granulosus* EGR-01664 contributes to the mechanism of parasitic immune evasion.

## 1. Introduction

Cystic echinococcosis (CE), a zoonotic disease endemic to natural foci, is caused by the metacestode larval stage of the tapeworm *E. granulosus* [1,2]. These larvae predominantly colonize the liver, lungs, and other organs in both humans and animals, resulting in substantial physiological impairment [3]. Protoscoleces, which are critical structures within hydatid cysts, demonstrate notable viability and proliferative potential [4]. Upon rupture of the cyst, the release of protoscoleces facilitates their dissemination into new host tissues, resulting in the formation of secondary cysts and exacerbation of the disease [5]. Therefore, comprehensive research into the biological characteristics of protoscoleces and their interactions with the host is essential for effective CE management. Studies have shown that the major egg antigens (MEAs) of Echinococcus have a typical α-crystal domain, which belongs to the HSP20 superfamily and contains multiple phosphorylation sites. It contains multiple phosphorylation sites, suggesting that it may be involved in the regulation of cell cycle, signal transduction, and growth and development through phosphorylation modification [6]. Based on the structural characteristics, MEAs may play an important role in the development of adult *E. granulosus* and its colonization in dogs, but the specific mechanism remains to be further elucidated. In terms of immune response, although there is currently no direct evidence of the immunogenicity of MEAs in dogs, studies have shown that Echinococcus infection can stimulate the host to produce a wide range of humoral and cellular immune responses, manifested as increased serum antibody levels and significant activation of Th1/Th2 cytokine responses [7]. It is worth noting that MEAs have significant genus, species, strain, and developmental stage specificity, which provides an important molecular basis for accurate diagnosis and targeted prevention and control of parasitic diseases [8], and they play a significant role in elucidating the host–parasite immune interaction network and the mechanisms underlying infection pathogenesis.

Adults of *E. granulosus* parasitize the small intestine of dogs. The eggs of *E granulosus* are produced during pregnancy and excreted with dog feces, becoming a source of infection for zoonotic hydatid disease [9]. When the intermediate host swallows the eggs, only a very small number of eggs can successfully hatch and develop into lesions in the host due to the lack of protection provided by the stratum corneum [10]. Therefore, the number and activity of eggs directly determine the success or failure of infection of intermediate hosts. Successfully hatched larvae will further develop into hydatid cysts (Eg-PSC) filled with cystic fluid and protoscoleces, eventually causing CE [11]. Despite the host’s ability to mount a specific immune response, the infection often persists for extended periods [12]. This persistence is primarily attributed to the parasite’s evolution of diverse immune evasion strategies that circumvent the host’s immune clearance mechanisms [13]. Within the intermediate host, the parasite penetrates the physical barrier of the cyst wall and secretes immunomodulatory factors, such as antigen B. This process alters the local microenvironment, promoting Th2-type immune polarization and regulatory cell responses, thereby inducing immune tolerance and facilitating the chronic progression of the infection [14,15]. Conversely, in the definitive host, the canine, the adult parasite encounters a robust mucosal immune response, characterized by IgE-mediated mast cell and eosinophil activation [4]. Although the helminths can temporarily evade the immune system through mechanisms such as surface antigen shedding, the infection is largely self-limiting and can confer a degree of acquired immune protection [16]. Currently, research on the immune interactions between helminths and their intermediate hosts is relatively comprehensive, encompassing areas such as liver fibrosis, immune evasion mechanisms, regulation of gut microbiota, and multi-level omics analyses [11,17,18]. In contrast, investigations into the immune interaction mechanisms in definitive hosts remain comparatively underdeveloped. Our previous study conducted a systematic analysis of the transcriptional expression profile of canine intestinal epithelial cells in response to protoscolex infection [19]. The current understanding of the immune response mechanisms in definitive hosts remains in its early stages. The key regulatory genes involved in the processes of infection, colonization, development, immune evasion, and parasite clearance have yet to be clearly defined. This knowledge gap not only impedes a comprehensive understanding of parasite–definitive host interactions but also substantially restricts the advancement of effective vaccines. Consequently, elucidating the immune regulatory mechanisms in definitive hosts is of substantial scientific value and holds significant potential for practical application.

Dendritic cells (DCs), recognized as the most potent professional antigen-presenting cells, serve a critical role in linking innate and adaptive immunity. as well as in modulating immune responses and tolerance [20]. Research indicates that Echinococcus can persist in the host for extended periods, a phenomenon closely associated with the immune tolerance induced by DCs [21,22]. In this study, antigens were extracted from experimental canines infected with protoscoleces (resulting in adult worm development) and isolated from primary cells derived from their intestinal Peyer’s patches. DCs within lymphocytes were isolated using magnetic bead sorting, and the protoscoleces antigens internalized by DCs were analyzed via mass spectrometry. Through genome-wide analysis of *E. granulosus*, we found a gene EGR-01664 (Major Egg Antigen, *E. g* MEA). Currently, the function of this protein remains unreported. This study aims to analyze the primary egg antigen genes of *E. granulosus* through bioinformatics, molecular cloning, recombinant protein expression, antibody preparation, and serological detection. We examined the protein distribution and transcription levels of EGR-01664. Additionally, we investigated the immunomodulatory effects of recombinant EGR-01664 (rEGR-01664) on canine peripheral blood mononuclear cells (PBMCs). The objective of this study was to evaluate the impacts of rEGR-01664 on the metabolic activity, polarization status, and inflammatory responses of canine PBMCs. These findings may help elucidate the immune interaction mechanisms between *E. granulosus* and its definitive host, while providing a theoretical basis for the development of CE vaccines.

## 2. Material and Methods

### 2.1. Parasites and Experimental Animals

The protoscoleces of *E. granulosus* used in this study were collected from a slaughterhouse in Shihezi City, Xinjiang Uygur Autonomous Region, China. The livers of donor sheep displayed typical cystic echinococcosis lesions, characterized by scattered round or oval cysts on the surface with diameters ranging from several centimeters to nearly occupying the entire hepatic lobe. The surfaces of *E. granulosus* cysts were rinsed repeatedly with sterile phosphate-buffered saline (PBS) to remove contaminants, then transferred to a sterile Petri dish. Cysts were punctured with a syringe to collect protoscolex-containing cyst fluid, which was allowed to stand at room temperature for 30–60 min to facilitate natural sedimentation of protoscoleces. After discarding the supernatant, the sediment was washed repeatedly and centrifuged 2–3 times in PBS supplemented with double antibiotics (penicillin–streptomycin). Protoscoleces were further purified by sequential filtration through 100-mesh and 400-mesh cell sieves. Their viability was evaluated using the trypan blue exclusion assay, and only those with intact morphology and >95% viability were used for subsequent experiments. The processed protoscoleces were then treated with Trizol reagent (Invitrogen, Shanghai, China). Concurrently, prior to conducting immunolocalization experiments, a subset of protoscoleces was fixed using a 4% paraformaldehyde (Nanjing Vazyme Biotech, Nanjing, China) solution. Adult samples were supplied by the State Key Laboratory of Sheep Genetic Improvement and Healthy Breeding. Female BALB/c mice (aged 6–8 weeks, weighing 22–25 g) were procured from the Experimental Animal Center of Xinjiang Medical University. Beagle canines were sourced from the Beagle canine Breeding Center of the Sichuan Musk Deer Research Institute.

### 2.2. Recombinant Plasmids, Sera, Cells, and Related Reagents

Plasmid pET-32a-EGR-01664, twenty-eight-day strobilated worms, infected canine serum, and peripheral blood mononuclear cells were derived from the State Key Laboratory of Sheep Genetic Improvement and Health Breeding. PCR-related reagents, *Escherichia coli* DH5α and BL21 (DE3) competent cells were purchased from Tiangen Biotech; pMD19-T vector, Taq DNA polymerase, T4 DNA ligase, restriction endonucleases (EcoR I, Xho I), and DNA Marker were purchased from Takara Biomedical Technology. Reagents and antibodies related to Western blot were purchased from Kangwei Century (Beijing, China). Reagents for protein induction, cell lysis, flow cytometry, and related secondary antibodies were obtained from Solarbio (Beijing, China).

### 2.3. Bioinformatics Analysis of EGR-01664

The amino acid sequence of the protein EGR-01664 (GenBank ID: 36337379) was obtained from the GenBank public database. Subsequent analysis of its physicochemical properties was conducted utilizing ProtParam. The hydrophilicity profile was evaluated through the Expasy ProtScale tool. Prediction of transmembrane domains was performed using the TMHMM Server version 2.0. Signal peptides and their cleavage sites, which provide insights into subcellular localization, were identified using SignalP version 6.0. Phosphorylation sites were predicted with NetPhos version 3.1, while N-glycosylation sites were identified using NetNGlyc version 4.1. Prediction of linear B-cell epitopes was carried out using ABCpred. The secondary structure was analyzed with SOPMA, and a tertiary structure model was constructed through homology modeling on the SWISS-MODEL platform.

### 2.4. Construction of Phylogenetic Tree

The amino acid sequence of the EGR-01664 protein, in FASTA format, was analyzed using the MEGA-11 software. A phylogenetic tree was subsequently constructed employing the Neighbor-Joining method. To ensure robustness, the Bootstrap test was conducted with 1000 replicates, culminating in the final construction of the phylogenetic tree for the EGR-01664 protein.

### 2.5. Design and Synthesis of Primers

Specific primers targeting the coding sequence (CDS) region of EGR-01664 from *E. granulosus* (GenBank accession number EUB63582.1) were designed and retrieved from GenBank. The design process utilized Primer Premier 5.0 software, incorporating an EcoR I restriction site in the forward primer and a Hind III restriction site in the reverse primer. The sequences of the primers are detailed in Table 1. Synthesis of the primers was conducted by Xinjiang Youkang Biotechnology Co., Ltd. (Youkang, China).

### 2.6. PCR Amplification of the Target Gene

RNA was isolated from frozen protoscoleces and adult samples utilizing an RNA extraction kit (Nanjing Vazyme Biotech, Nanjing, China). The concentration of the extracted RNA was quantified using a NanoDrop2000c ultra-micro spectrophotometer (Thermo Fisher Scientific). Reverse transcription was performed using the complementary DNA (cDNA) synthesis kit (Nanjing, Vazyme Biotech, Nanjing, China), which was subsequently used as a template for further analysis. The CDS region of the EGR-01664 gene was amplified using the primers specified in Table 1. The PCR was performed in a 20 μL reaction system containing 10 μL of 2× Taq PCR Master Mix II, 0.5 μL each of forward and reverse primers, 1 μL of cDNA template, and 8 μL of dd H_2_O. The amplification protocol consisted of an initial denaturation step at 95 °C for 5 min, followed by 35 cycles of denaturation at 95 °C for 30 s, annealing at 60 °C for 30 s, and extension at 72 °C for 62 s, with a final extension at 72 °C for 5 min. Amplified products were stored at 4 °C and subsequently analyzed by 1.5% agarose gel electrophoresis.

### 2.7. Construction and Verification of Cloning Plasmid pMD19-T-EGR-01664

The target fragment was inserted into the pMD19-T vector (Takara, Beijing, China), with the resulting plasmid designated pMD19-T-EGR-01664. This recombinant plasmid was subsequently introduced into *E. coli* DH5α competent cells (Tiangen, Beijing, China) via transformation. The transformed cells were plated onto LB agar containing Ampicillin (100 μg/mL, Amp) and incubated overnight at 37 °C. Individual colonies were selected and inoculated into 1 mL of LB liquid medium supplemented with Amp, followed by incubation at 37 °C with shaking at 180 rpm for 4 h. The bacterial cultures were then subjected to PCR verification, utilizing the same reaction conditions as previously described. PCR-positive cultures were preserved in glycerol stocks, and the recombinant plasmid was extracted. The identity of the extracted plasmid was confirmed through double digestion with EcoR I and Hind III. The double digestion was performed in a 20 μL reaction mixture containing 7 μL of pMD19-T-EGR-01664 plasmid, 2 μL of 10× QuickCut Green Buffer, 1 μL each of the restriction enzymes EcoR I and Hind III, and nuclease-free water to a final volume of 20 μL. The digestion products were then resolved on a 1.5% agarose gel. Concurrently, the recombinant plasmid was submitted to Bioengineering Co., Ltd. (Youkang, China) for bidirectional sequencing verification.

### 2.8. Construction and Verification of Expression Plasmid pET32a-EGR-01664

The pET32a vector (Novagen, Darmstadt, Germany) and the cloning plasmid pMD19-T-EGR-01664 were subjected to EcoR I and Hind III double digestion. After the target DNA fragment was separated by agarose gel electrophoresis, the target band was excised under an ultraviolet lamp. Subsequently, an agarose gel recovery kit (Tiangen, Beijing, China) was used to perform gel dissolution, purification, and elution in sequence, and finally, high-purity target DNA fragments was obtained. The recombinant plasmid pET32a-EGR-01664 was assembled by ligating the pET32a vector and the *EGR-01664* gene fragment at 4 °C overnight. Following transformation into *DH5α* competent cells and overnight incubation at 37 °C, single colonies were inoculated into 1 mL of LB liquid medium supplemented with ampicillin. These cultures were incubated for 4 h at 37 °C and then screened by PCR (Table 1). From the positive clones identified, the plasmid was extracted and confirmed by double digestion, employing the same conditions as previously described. Successful identification was confirmed via 1.0% agarose gel electrophoresis. The recombinant plasmid pET32a-EGR-01664 was subsequently sent to Bioengineering Co., Ltd. for sequencing.

### 2.9. Expression, Purification, and Renaturation of rEGR-01664 Protein

The recombinant plasmid pET32a-EGR-01664 was expressed in *E. coli* grown in ampicillin-LB medium. Upon reaching an OD_600_ of 0.6–0.8, protein expression was induced with 1 mmol/L IPTG at 37 °C with shaking (180 rpm). Aliquots collected at 2, 4, 6, and 8 h post-induction were analyzed by SDS-PAGE to determine the optimal induction time. For protein purification, the bacterial cells from the optimal induction were harvested by centrifugation (10,000 rpm, 10 min, 4 °C). The pellet was resuspended in Tris-HCl buffer (50 mmol/L, pH 8.5), and the hypotonic environment of this buffer was used for osmotic lysis of the cells, followed by incubation at 4 °C overnight. The suspension was then subjected to five freeze–thaw cycles in liquid nitrogen, followed by ultrasonication. After centrifugation, equal volumes (80 μL) of the supernatant and pellet fractions were collected separately, and 20 μL of upper and lower buffers were added to each fraction and mixed well. Subsequently, 16 μL of the above mixed sample was taken for SDS-PAGE to detect the expression of the recombinant protein. After loading the disrupted bacterial pellet onto the column, the bottom of the column was sealed and incubated at 4 °C for 1 h to allow the target protein to bind to the resin. Subsequently, the outlet was opened to collect the flow-through. The column was sequentially washed with 10 column volumes of inclusion body protein A buffer (Binding buffer) to remove contaminating proteins, followed by elution of the target protein with 5 column volumes of buffer B (Elution buffer). The first 6–8 drops of dead volume were discarded, and the effective eluate fractions were collected. The eluate was transferred into dialysis bags pre-treated with 2% sodium bicarbonate and 1 mmol/L EDTA (pH 8.0), and dialyzed sequentially against a urea gradient from 8 mmol/L to 1 mmol/L and then against dd H_2_O, with each dialysis step lasting 8 h. Finally, the protein was concentrated by sucrose addition. The lipopolysaccharides from the recombinant proteins were detoxified using the ToxinEraserTM Endo-toxin Removal kit (GenScript, Nanjing, China). The concentration of the recombinant protein was quantified utilizing a NanoDrop 2000c ultra-micro spectrophotometer (Thermo Fisher Scientific). The sample was subsequently stored at −80 °C. The efficacy of the purification process was assessed via 12% SDS-PAGE analysis.

### 2.10. Identification of the Purified Protein by Western Blot

The immunogenicity of the refolded rEGR-01664 protein was assessed by Western blot. The protein was first resolved by SDS-PAGE and transferred onto a polyvinylidene fluoride (PVDF) membrane (Roche, Germany). The membrane was then blocked with 5% skimmed milk for 2 h at room temperature and washed three times with TBST. Subsequently, it was incubated with a primary antibody (positive serum from *E. granulosus*-infected canines; 1:100 dilution) and then with an HRP-conjugated rabbit anti-canine IgG secondary antibody (Solarbio, Beijing, China; 1:2000 dilution). Following antibody incubation, the HRP-DAB substrate chromogenic solution was prepared and applied to the membrane in the dark for color development, after which photographs were taken.

### 2.11. Preparation of Polyclonal Antibody Against Recombinant Protein and Determination of Antibody Titer

Female BALB/c mice (*n* = 6 per group) were randomly divided into three groups: one immunized with 50 μg of purified rEGR-01664 protein emulsified in Freund’s adjuvant (1:1 ratio), and a control group administered with an equal volume of PBS [23]. This mixture was subjected to overnight agitation at low temperature to ensure thorough mixing. The PBS control group received 200 μL of PBS via injection. Mice were immunized via subcutaneous injection on the back, with a total of three administrations at 7-day intervals. At 14 days post-final immunization, blood was collected from the orbital sinus, and the resulting serum was stored at −80 °C. Concurrently, normal rat serum was isolated to serve as a negative control.

To determine antibody titers, an ELISA was performed according to the manufacturer’s instructions (Solarbio, Beijing, China). The recombinant protein served as the capture antigen for the assay. After blocking, serially diluted mouse sera were applied as the primary antibody, respectively. Subsequently, an HRP-labeled goat anti-mouse IgG secondary antibody (1:1000; Solarbio, Beijing, China) was added. The OD_450_ was read after substrate addition, and the endpoint titer was calculated as the maximum dilution factor at which the OD_450_ value was at least two-fold higher than that of the negative control.

### 2.12. Immunofluorescence Localization of Recombinant Proteins

Fresh protoscoleces were cultured in a complete medium consisting of RPMI 1640 supplemented with 2% penicillin–streptomycin (Sigma-Aldrich, St. Louis, MO, USA) and 10% fetal bovine serum albumin (BSA; Hyclone, Logan, UT, USA). The vitality and morphology of the protoscoleces were monitored. Protoscoleces with intact cyst walls, distinct internal structures, and active flame cell activity, as observed under a microscope, were selected for subsequent IFA. The initial medium was discarded, and the protoscoleces were washed three times with 1× PBS, each wash lasting 5 min. The protoscoleces were then fixed with precooled 4% paraformaldehyde overnight, after which the fixative was removed. Samples were subjected to three 5 min washes with PBST. Next, cells were permeabilized using 1% Triton X-100 (Biofroxx, Einhausen, Germany) for 1 h, followed by three 5 min rinses with PBS. After blocking with BSA at 37 °C for 2 h, cells underwent three 5 min washes with PBST. Mouse positive and negative sera, diluted 1:100 in PBST, were incubated overnight at 4 °C and then rinsed five times with PBST (5 min per wash). Goat anti-mouse IgG-FITC antibody (Solarbio, Beijing, China) was diluted 1:2000 in PBST and incubated in the dark at 37 °C for 2 h. Finally, cells were washed three times with PBST in the dark, with each wash lasting 10 min. Finally, PI staining solution was applied for 10–15 min, and a total of 20 μL of the insect suspension was placed on a slide for analysis (Nikon, Tokyo, Japan).

### 2.13. Transcription Level Analysis of EGR-01664 Gene at Different Developmental Stages of Worms

qPCR was performed to analyze EGR-01664 expression in *E. granulosus* protoscoleces and 28-day strobilated worms cDNA, with β-actin as the reference gene (primers in Table 1). Reactions (20 μL total volume) contained 10 μL SYBR GREEN MIX, 1 μL cDNA, 1 μL each primer, and 7 μL nuclease-free water. The protocol comprised an initial 95 °C for 2 min, followed by 45 cycles of 95 °C for 15 s, 60 °C for 30 s, and 68 °C for 10 s. The results were analyzed using the 2^−ΔΔCt^ method and shown as the relative expression to the gene of strobilated worm.

### 2.14. Isolation and In Vitro Culture of Canine PBMCs

In a superclean bench, the collected canine peripheral blood was transferred to a centrifuge tube. The procedure adhered strictly to the standard protocol outlined in the canine peripheral blood lymphocyte isolation kit (Solarbio, Beijing, China). The detailed steps are as follows: Initially, an equal volume of diluent was added to the blood sample. After gentle mixing, the separation solution was pre-added to a new 50 mL centrifuge tube. Subsequently, the diluted blood was carefully layered along the tube wall, ensuring that the combined volume of the separation fluid and blood did not exceed two-thirds of the centrifuge tube’s capacity. Owing to the differences in liquid density, the diluted blood naturally stratified above the separation solution. The sample was centrifuged at 300 rpm for 30 min at 4 °C. Post-centrifugation, the liquid in the tube exhibited distinct stratification, comprising, from bottom to top, the red blood cell layer, separation liquid layer, lymphocyte layer, and diluent layer. Lymphocytes were collected, washed in complete RPMI 1640 medium, and pelleted by centrifugation. After red blood cell lysis (on ice, 10 min) and subsequent centrifugation, the cells were washed thrice with PBS. The purified lymphocytes were then resuspended in 1× PBS for counting and subsequently cultured at the required density in complete RPMI 1640 medium at 37 °C with 5% CO_2_.

### 2.15. The Effect of EGR-01664 Gene on the Proliferation of Canine PBMCs

Cells were seeded into each well of 96-well cell culture plates at densities of 1, 2, 3, 4, 5, and 6 × 10^5^ cells. Three replicates were performed for each condition. Following the addition of CCK8 reagent (Tiangen, Beijing, China) and a 6 h incubation period, the OD values were recorded for each well. These OD values, in conjunction with the corresponding cell numbers, were used to establish a standard curve. Subsequently, cells were seeded in a new 96-well plate at a density of 5 × 10^5^ cells per well and assigned to five treatment groups (*n* = 3 wells per group). The groups were treated with 1× PBS (control) or the recombinant protein at final concentrations of 5, 10, 20, or 40 μg/mL as described by [24]. After a 24 h co-culture period, CCK8 reagent was added, and following an additional 6 h incubation, the OD values were measured. The number of canine PBMCs was subsequently calculated using the established standard curve.

### 2.16. Nitric Oxide (NO) Production Assay

Fresh canine PBMCs were plated in 24-well plates at a density of 1 × 10^6^ cells per well in a final volume of 100 μL. Recombinant proteins were added to achieve final concentrations of 5, 10, 20, and 40 μg/mL, or equal volumes of PBS were added as controls. Cells were incubated at 37 °C for 24 h, with each condition run in triplicate. The concentration of nitric oxide in the supernatant was subsequently determined using a dedicated NO detection kit (AIDISHENG, Jiangshu, China).

### 2.17. Detection of Cytokines Level

Canine PBMCs, at a density of 5 × 10^5^ cells per mL per well, were seeded into 24-well plates and incubated at 37 °C with 5% CO_2_. Upon reaching 80% density, the cells were co-cultured with various concentrations of rEGR-01664 (5, 10, 20, and 40 μg/mL) and equal volumes of PBS for a duration of 24 h. Subsequently, the co-cultured canine PBMCs were harvested, and total RNA was extracted and followed by reverse transcription into cDNA. Specific primers for canine TGF-β, IL-10, IL-17A, IFN-γ, Bcl-2, GSDMD, and Bax genes were designed and synthesized, with the primer sequences detailed in Table 1. The relative expression of target genes in canine PBMCs was quantified by qPCR, normalized to the endogenous control β-actin, and calculated using the 2^−ΔΔCt^ method. All treatments were performed in triplicate, and the effects of recombinant protein on cytokine/gene expression were assessed by statistical analysis.

### 2.18. Statistical Data Analysis

All statistical analyses were performed using GraphPad Prism software (version 9.5). Data are reported as the mean ± standard error (SEM). Statistical significance was defined as * *p* < 0.05, ** *p* < 0.01, *** *p* < 0.001, and **** *p* < 0.0001, with *p* > 0.05 considered not significant (ns).

## 3. Results and Analysis

### 3.1. Bioinformatic Analysis of EGR-01664

**Figure 1:**
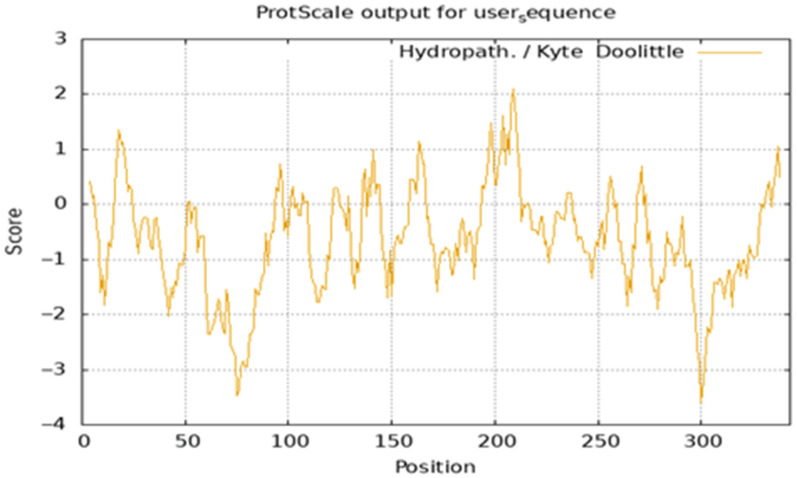
Hydrophilic analysis of EGR-01664 protein.

The EGR-01664 protein has a molecular formula of C_3074_H_5118_N_1032_O_1268_S_290_, with a relative molecular mass of 38,097.05 and a theoretical isoelectric point (pI) of 6.88. It is predicted to be a stable protein, lacking both a signal peptide and transmembrane domains (Table 2). Additionally, the protein exhibits overall hydrophilicity, with a grand average of hydropathicity (GRAVY) of −0.627 (Figure 1).

**Figure 2:**
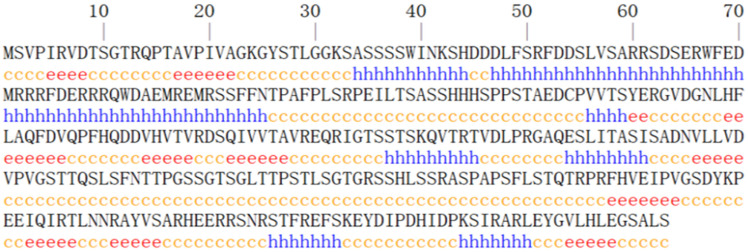
Secondary structure prediction of EGR-01664 protein. h stands for alpha helix; e stands for extended chain; c means random curling.

**Figure 3:**
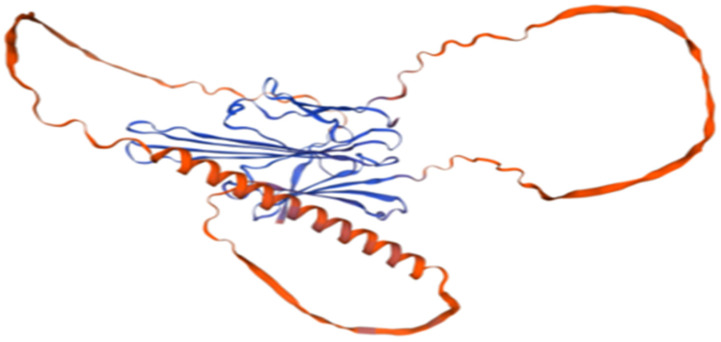
Predicted tertiary structure predictions for EGR-01664.

The secondary structure of the EGR-01664 protein was predicted utilizing the SOPMA I software. The analysis revealed that 95 residues (27.70%) were classified as α-helices (Hh), 58 residues (16.91%) as β-sheets (Ee), and 190 residues (55.39%) as random coils (Cc); refer to Figure 2. Subsequently, the three-dimensional structural model was generated using the SWISS-MODEL online platform; refer to Figure 3. The resulting model predominantly comprised α-helices and random coils, corroborating the findings of the secondary structure prediction.

### 3.2. Phylogenetic Tree of EGR-01664 Gene

**Figure 4:**
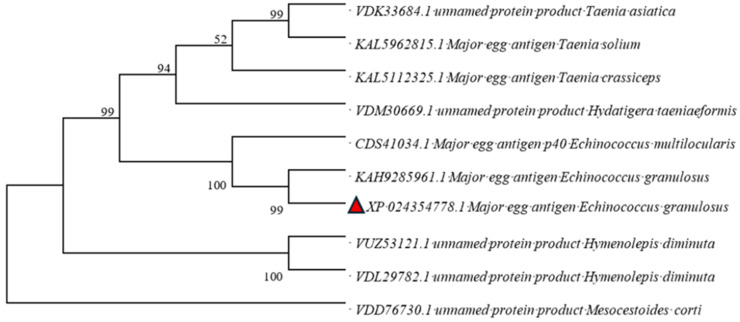
Phylogenetic tree of the EGR-01664 protein. 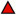 Marked as the EGR-01664 protein sequence in this study.

The phylogenetic tree depicted in Figure 4, which was constructed utilizing the multiple sequence alignment results from MEGA 11 software, demonstrates that EGR-01664 and *Echinococcus multilocularis* (*E. multilocularis)* are grouped within the same clade, suggesting a close evolutionary relationship. In contrast, Taenia solium and Taenia crassirollis are positioned on separate branches, indicating a more distant phylogenetic relationship.

### 3.3. PCR Amplification of EGR-01664 Gene

**Figure 5:**
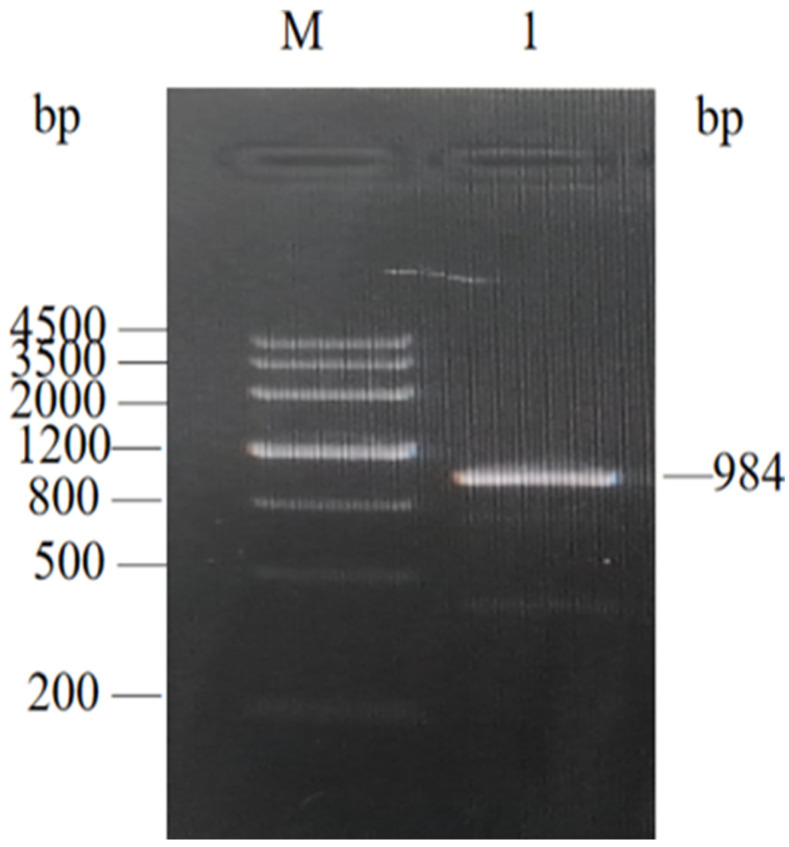
PCR amplification of *EGR-01664* gene of *E. granulosus.* M: DNA Marker; 1: PCR amplification product of *EGR-01664* gene.

The coding sequence (CDS) of EGR-01664 comprised 984 base pairs (bp). PCR amplification was conducted using specifically designed primers. The resultant PCR products were subjected to analysis via agarose gel electrophoresis at a concentration of 15 g/L. The electrophoresis results indicated the presence of a distinct amplified band approximately at 1000 bp, aligning with the anticipated size of 984 bp (Figure 5). Subsequent sequencing confirmed the accuracy of the nucleic acid sequence, rendering it suitable for use in the construction of a recombinant plasmid in subsequent experimental phases.

### 3.4. Identification of Recombinant Plasmid pET32a-EGR-01664

**Figure 6:**
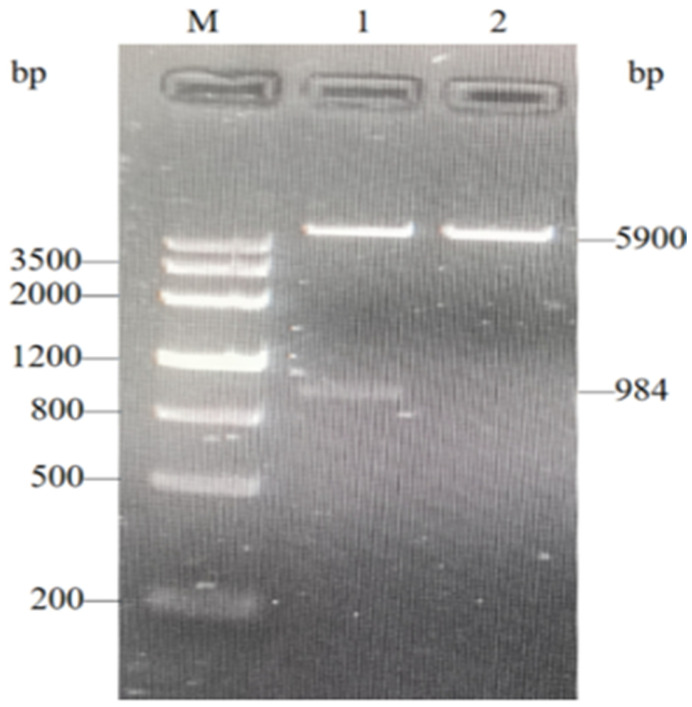
Identification of recombinant plasmid pET32a-EGR-01664 by double restriction enzyme digestion. M: DNA Marker. 1: Double-enzyme digestion products of pET32a-EGR-01664. 2: Recombinant plasmid pET32a-EGR-01664 plasmid.

The recombinant plasmid was characterized through double enzyme digestion using EcoR I and Hind III, followed by agarose gel electrophoresis. This analysis revealed fragments approximately 5000 bp and 984 bp in length (Figure 6), corresponding to the expected sizes of the expression vector pET-32a and the target gene EGR-01664, respectively.

### 3.5. Expression, Purification, and Identification of Recombinant Protein EGR-01664

**Figure 7:**
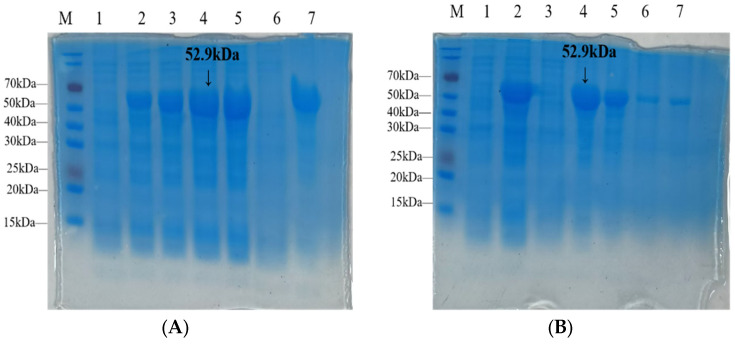
Induced expression and purification of recombinant strain pET32a-EGR-01664/BL21. (**A**). M: protein molecular quality standards; 1: pET32a-EGR-01664/BL21 recombinant bacteria without IPTG oscillation culture; 2–5: pET32a-EGR-01664/BL21 recombinant bacteria induced by IPTG for 2, 4, 6, and 8 h; 6: supernatant after cell disruption; 7: sedimentation after cell crushing. (**B**). M: Protein molecular weight standard; 1: Uninduced pET32a-EGR-01664/BL21; 2: IPTG induction for 6 h; 3: crushing supernatant; 4: fragmentation precipitation; 5: effluent; 6: A liquid elution (Binding buffer); 7: B liquid elution (Elution buffer).

The recombinant plasmid pET32a-EGR-01664 was introduced into *E. coli BL21 (DE3)* competent cells, where it was subsequently induced to express the rEGR-01664 protein in a soluble form. The anticipated molecular weight of the expressed protein was approximately 52.9 kDa, as depicted in Figure 7A. The purification outcomes for the rEGR-01664 protein are presented in Figure 7B. Following nickel ion affinity chromatography, the protein product was eluted using Solution B.

### 3.6. Western Blot Detection of Recombinant Protein

**Figure 8:**
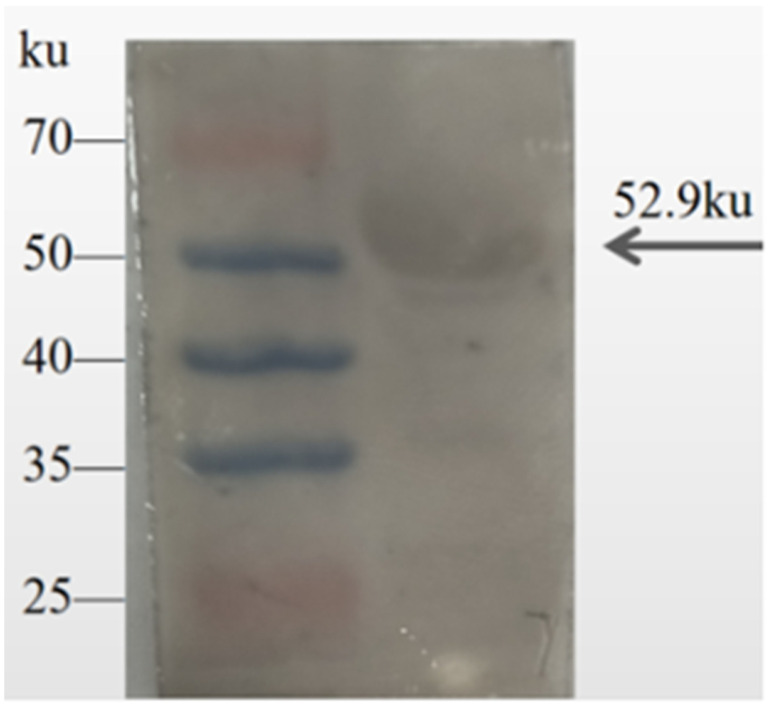
Western blot identification of rEGR-01664.

The primary antibody (canine positive serum) and secondary antibody (HRP-conjugated goat anti-dog IgG) were applied at dilutions of 1:400 and 1:4000, respectively. The specific band corresponding to rEGR-01664 was observed at 52.9 kDa. These findings demonstrate that the recombinant protein was successfully identified, suggesting it possesses strong antigenic properties (Figure 8).

### 3.7. Titer Determination of rEGR-01664 Polyclonal Antibody

**Figure 9:**
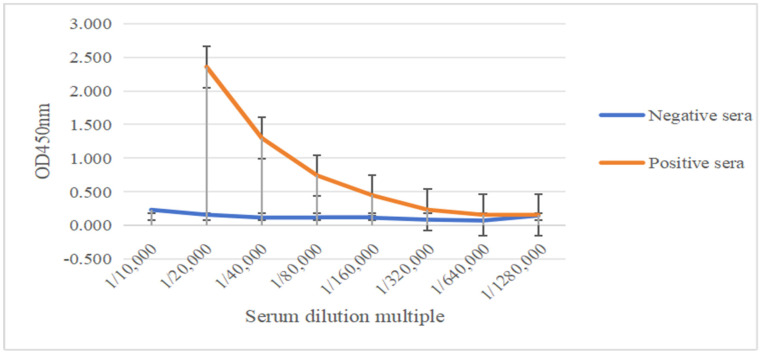
ELISA detection of the titer of polyclonal antibody against recombinant protein rEGR-01664 in mice.

The titer of rEGR-01664 mouse polyclonal antiserum was assessed using an indirect ELISA. As illustrated in Figure 9, upon dilution of the rEGR-01664 positive serum to 1:320,000, the OD value remained twice as high as that of the negative control serum. Consequently, the titer of the positive serum was determined to be 1:320,000. No specific antibody response was observed in the serum of the negative control group, underscoring the reliability of the experimental results. These findings suggest that the immune recombinant protein effectively stimulated the production of higher antibody titers in mice, thereby enhancing the credibility of the results.

### 3.8. Localization of rEGR-01664 Protein Antigen in Protoscoleces and Adults

**Figure 10:**
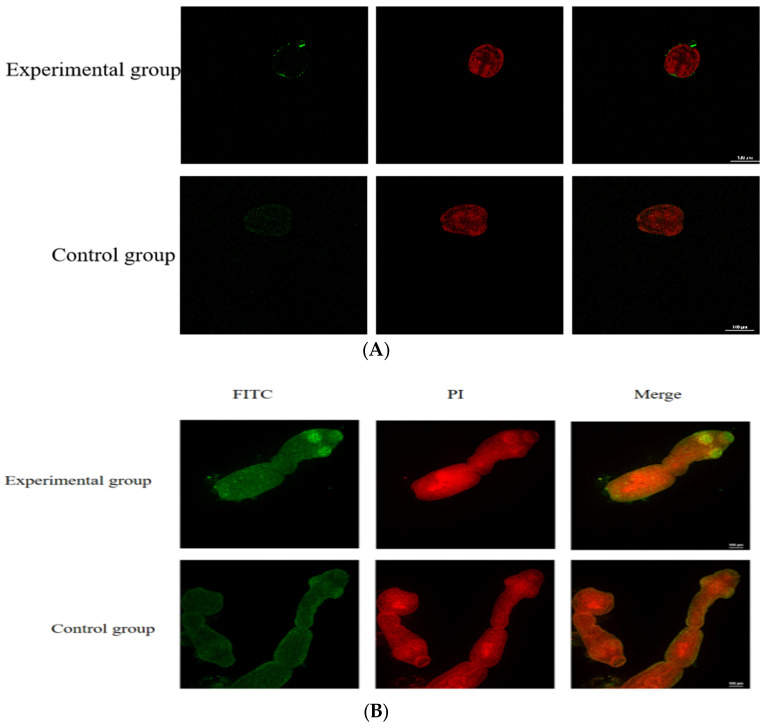
Tissue localization of EGR-01664 protein in (**A**) protocariae and (**B**) adult worms.

The immunofluorescence localization of *E. granulosus* protoscoleces and adult specimens was investigated utilizing a mouse anti-rEGR-01664 polyclonal antibody as the primary antibody, followed by a FITC-labeled goat anti-mouse IgG as the secondary antibody. The findings (Figure 10A) indicated a limited distribution of the target protein on the surface layer of the protoscoleces within the experimental group, whereas no such distribution was observed in the negative control group. The EGR-01664 protein was predominantly localized in the mouth hook region of protoscoleces that had not undergone eversion. In adult specimens, the protein was primarily concentrated in the head section and certain parenchymal tissues (Figure 10B).

### 3.9. The Differential Expression of EGR-01664 Gene in Different Developmental Stages of Insects

**Figure 11:**
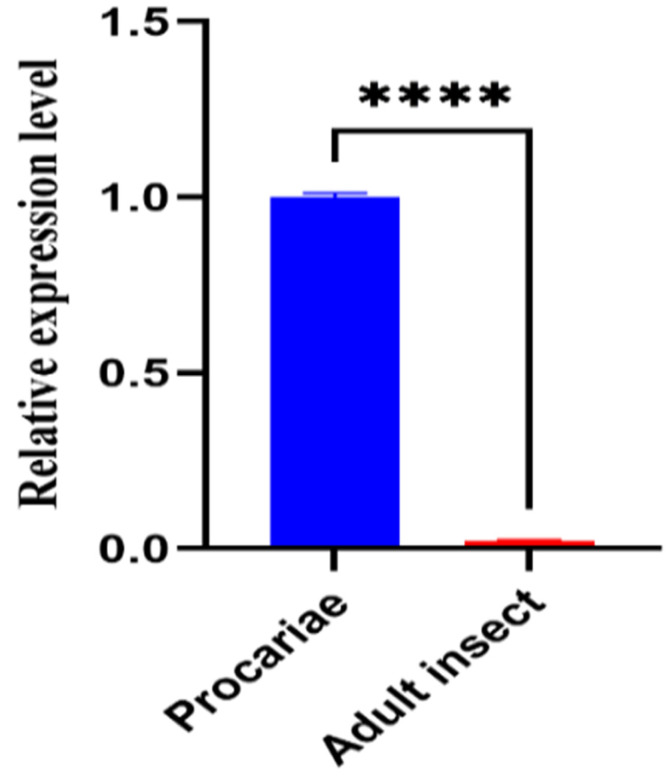
Analysis of the transcriptional levels of the *E. granulosus EGR-01664* gene at different developmental stages. **** *p* < 0.0001.

The expression profile of the EGR-01664 gene across various developmental stages of *E. granulosus* was examined utilizing qPCR methodology. As depicted in Figure 11, qPCR analysis detected EGR-01664 mRNA expression in both protoscoleces and adult worms of *E. granulosus*. The expression level was markedly higher in protoscoleces than in adult worms (*p* < 0.0001).

### 3.10. rEGR-01664 Promotes the Proliferation of Canine PBMCs

**Figure 12:**
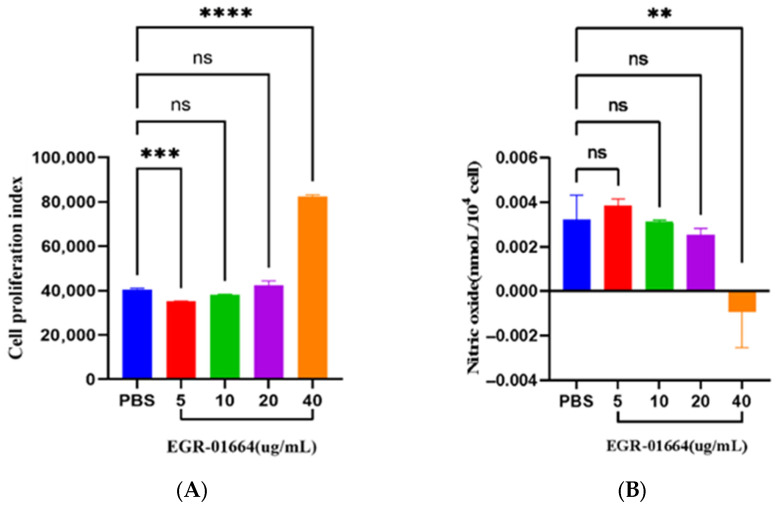
(**A**) The effect of EGR-01664 on the proliferation of PBMCs in canines. (**B**) The effect of rEGR-01664 on NO in canine PBMCs. ns *p* ≥ 0.05, ** *p* < 0.01, *** *p* < 0.001, **** *p* < 0.0001.

The CCK-8 assay showed that rEGR-01664 stimulated the proliferation of canine PBMCs in a dose-dependent manner (Figure 12A). Cell proliferation rates increased with escalating concentrations of the protein, with the 40 μg/mL group showing a particularly significant effect compared to the PBS control (*p* < 0.0001).

### 3.11. rEGR-01664 Inhibited the Ability of Canine PBMCs to Produce Nitric Oxide

A total nitric oxide assay kit was employed to quantify NO levels in canine PBMCs subjected to varying concentrations of rEGR-01664. The findings indicated that rEGR-01664 modulated NO production in a dose-dependent fashion. A marked suppression of NO production was observed in the group treated with 40 μg/mL rEGR-01664 relative to the PBS control (Figure 12B).

### 3.12. Analysis of Cytokine Production by rEGR-01664

**Figure 13:**
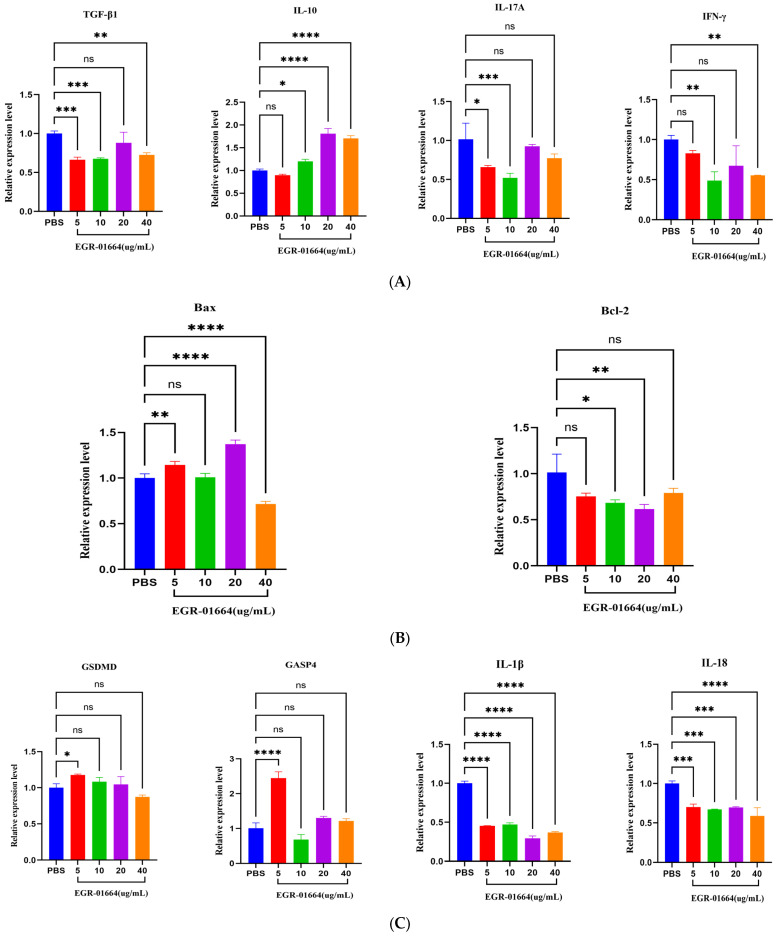
(**A**) Effects of rEGR-01664 on the transcription levels of cytokines in canine PBMCs. (**B**) The effect of rEGR-01664 on the transcription level of apoptosis-related genes in canine PBMCs. (**C**) The effect of rEGR-01664 on the transcription level of pyroptosis-related genes in canine PBMCs. Compared with control, ns and * *p* ≥ 0.05, ** *p* < 0.01, *** *p* < 0.001, **** *p* < 0.0001.

The qPCR analysis demonstrated that the protein markedly suppressed the expression of several pro-inflammatory factors, including IL-17A, IFN-γ, IL-1β, and IL-18, as well as the anti-apoptotic factor Bcl-2, as illustrated in Figure 13. Concurrently, it enhanced the expression of the anti-inflammatory factor IL-10 and the pro-apoptotic/pyroptosis factors Bax, GSDMD, and CASP4 (Figure 13). Notably, low concentrations (5 and 10 μg/mL) significantly inhibited the expression of TGF-β1 (Figure 13). It is important to highlight that IL-1β and IL-18 were significantly inhibited across all concentrations (*p* < 0.0001), whereas the enhancing effect on Bax and GSDMD observed at a concentration of 40 μg/mL was not present (Figure 13).

### 3.13. Descriptive Results of the Study

In summary, EGR-01664 antigen is immunogenic, regulated by development, and located in the key parasitic structure of *E. granulosus*. Its recombinant form can directly regulate the function of host immune cells by promoting proliferation, inhibiting inflammatory response and dysregulated cell death pathways, suggesting its role in immune escape.

## 4. Discussion

### 4.1. Research Background and the Potential Significance of EGR-01664

China is among the countries most severely threatened by *E. granulosus*, with significant detrimental impacts reported [24,25]. The principal egg antigen of these parasites serves as a crucial molecule mediating the interaction between parasite development and host immune responses. Research on this antigen is pivotal not only for advancing fundamental understanding of pathogenic biology but also for its core significance in vaccine development, diagnostic technologies, and strategies to block transmission [26]. Major egg antigens have garnered substantial attention in the context of parasite diagnosis and vaccine development. In the study conducted by [27], the major egg antigen Smp40 was identified as a highly promising candidate in Schistosoma mansoni, due to its potential to mitigate collagen deposition, reduce fibrosis, and inhibit granuloma formation. Furthermore, studies have confirmed that the principal major egg antigen (*Em MEA*) of *E. multilocularis* could specifically react with the serum of infected mice [28].

### 4.2. Bioinformatics Characteristics of EGR-01664

The predicted EGR-01664 protein is characterized by 54 phosphorylation sites and 2 N-glycosylation modification sites, suggesting potential alterations in its structure and conformation. Subcellular localization predictions indicate the absence of both a signal peptide and a transmembrane domain in the protein. Furthermore, B-cell linear epitope predictions reveal that EGR-01664 possesses multiple potential linear epitopes, suggesting a high potential for immunogenicity. Predictions of the secondary and tertiary structures indicate that the primary structural elements of the protein include α-helices, β-sheets, and random coils. The α-helices, which form the core framework, confer structural stability, although their regular conformation presents challenges in forming effective antigenic epitopes [29]. In contrast, β-sheets play a role in protein recognition and assembly, contributing to overall conformational stability [30]. The random coils, located on the surface, are conducive to forming effective antigenic epitopes due to their loose structure and high flexibility [31], aligning with the protein’s favorable antigenicity predictions.

### 4.3. Expression and Immunogenicity Verification of EGR-01664 Protein

In this study, the recombinant antigen rEGR-01664 from *E. granulosus* was successfully cloned, expressed, and purified. When used to immunize mice, it induced a robust serum antibody response. Western blot analysis confirmed the immunogenicity of rEGR-01664, as evidenced by its specific reactivity with sera from *E. granulosus*-infected canines. Additionally, indirect immunofluorescence assay (IFA) revealed that the EGR-01664 protein is predominantly localized to the rostellar hook region of protoscoleces and the parenchyma of adult worms, implying a potential role in parasite invasion and colonization. Collectively, these results suggest that rEGR-01664 represents a promising candidate antigen for developing vaccines against *E. granulosus* infection in definitive hosts.

### 4.4. Immunomodulatory Effects of rEGR-01664 on Canine PBMCs

Peripheral blood mononuclear cells (PBMCs), comprising lymphocytes, monocytes, and dendritic cells, are essential for investigating immune function [32]. This study investigates the impact of the recombinant protein rEGR-01664 on the immune function of canine PBMCs, focusing on cell proliferation and NO production. This research provides a foundation for elucidating the immune mechanisms between *E*. *granulosus* and its host. The CCK-8 assay is extensively utilized for screening anti-malarial, toxoplasmosis, and ciliate drugs by assessing the metabolic activity of parasites or host cells [33]. Protein analysis serves as a vital supplementary method, enhancing the understanding of proliferation and inhibition mechanisms, validating the reliability of CCK-8 results, and providing deeper insights into cell states [34]. The study observed that increasing concentrations of rEGR-01664 protein stimulation correlated with an upward trend in the number of PBMCs. Among the tested concentrations, the 40 μg/mL group demonstrated a particularly significant effect on cell proliferation (*p* < 0.0001). NO is a pleiotropic signaling molecule that regulates diverse physiological processes, including cytotoxicity and endocrine hormone release, and is implicated in the pathogenesis of numerous diseases [35]. NO serves as the primary effector molecule for macrophages in eradicating intracellular pathogens. Previous studies have indicated that Leishmania infection in host macrophages can suppress the expression of inducible nitric oxide synthase (iNOS), thereby reducing NO production and evading the host’s immune clearance [36]. In the present study, it was observed that the EGR-01664 protein of *E. granulosus* could inhibit NO production in canine PBMCs in a dose-dependent manner.

Cytokines secreted by Th1 and Th2 immune and inflammatory responses are crucial in combating parasitic infections [37]. Thelper cells (Th) differentiate into various subsets of effector T cells upon antigen stimulation, with each subset characterized by distinct cytokine profiles that direct specific immune regulatory pathways [38]. Th1 cytokines contribute to parasite clearance, whereas Th2 cytokines are associated with parasite immune evasion [39,40]. Th1 cells secrete IFN-γ, which enhances macrophage activation and their antigen-presenting capabilities. Th17 cells secrete IL-17A and, along with Treg cells, are implicated in the development of chronic inflammatory responses, such as those observed in CE [40,41]. Treg cells secrete IL-10 and TGF-β1, with TGF-β1 potentially signaling the suppression of immune responses and inhibiting various immune cell functions [38]. Studies have shown that the recombinant proteins EgANXB18 and EgANXB20 of *E. granulosus* can regulate the immune response between parasites and hosts by affecting the expression of Th1, Th2, Th17, and/or Treg cytokines [42,43]. We quantified the expression of key cytokines, including the Th1 cytokine IFN-γ, the Th2 cytokine IL-10, the pro-inflammatory cytokine IL-17A, and the Treg cytokine TGF-β1. The results indicated that rEGR-01664 markedly enhanced the secretion of IL-10 while significantly suppressing the relative mRNA level of IFN-γ in canine PBMCs. No significant effects were observed on the other cytokines measured. Notably, IL-10 and TGF-β1, primarily produced by Treg cells, are widely recognized for their immunosuppressive functions [44]. IFN-γ activates macrophages through the JAK-STAT signaling pathway and induces them to produce a large amount of NO (produced by inducible nitric oxide synthase iNOs) and reactive oxygen species [45]. The immunomodulatory effects of rEGR-01664, characterized by elevated IL-10 and suppressed IFN-γ, point to its potential role in the immune evasion strategies of *E. granulosus*. Although TGF-β1 production was not enhanced in our experimental model, the significant induction of IL-10 alone may be sufficient to drive a suppressive response. Further investigation is needed to fully elucidate the mechanistic basis of this selective cytokine induction and it in vivo relevance.

### 4.5. Regulatory Effects of rEGR-01664 on Cell Apoptosis and Pyroptosis

Apoptosis serves as a crucial regulatory mechanism in parasitic infections, governed by the intricate balance between pro-apoptotic and anti-apoptotic proteins. Central to this apoptotic pathway are the Bcl-2 family proteins (B-cell lymphoma-2 protein family), which play a pivotal role [46]. The Bcl-2 protein inhibits apoptosis by preventing Bax-mediated mitochondrial outer membrane permeability (MOMP) [47]. The relative expression levels of Bax and Bcl-2 are critical in determining the apoptotic outcome: elevated Bax levels enhance apoptosis, whereas increased Bcl-2 levels suppress it [48]. In the present study, rEGR-01664 markedly induced the expression of the pro-apoptotic factor Bax, with the maximum effect observed at a concentration of 20 μg/mL, demonstrating significant difference from the PBS control (*p* < 0.0001). Concurrently, the expression of the anti-apoptotic factor Bcl-2 was markedly downregulated, reaching its lowest level at the same concentration, also significantly different from the PBS control group (*p* < 0.01). These results suggest that rEGR-01664 may reduce the expression of Bcl-2 by inhibiting the transcriptional activity of STAT3 or CREB. At the same time, it promotes the expression of Bax by activating p53, thereby synergistically enhancing mitochondrial membrane permeability and ultimately inducing apoptosis of host effector immune cells. Further studies are needed to elucidate its molecular pathways and regulatory mechanisms through functional experiments.

Pyroptosis is a programmed, pro-inflammatory cell death mediated by inflammatory caspases (such as caspase-1/4/5/11), which cleave gasdermin D to induce plasma membrane pore formation [49]. CASP1, a cysteine protease, is pivotal in the activation of classical inflammasomes, which subsequently initiate the inflammatory response by cleaving and facilitating the maturation of interleukin-1β (IL-1β) and interleukin-18 (IL-18) [50]. In contrast, the non-classical inflammasome pathway is primarily mediated by Caspase-4 (CASP4), which directly recognizes and binds to lipopolysaccharide (LPS) independently of the classical inflammasome components [51]. In certain cell types, such as monocytes and macrophages, CASP4 can partially substitute for the function of CASP1. Gasdermin D (GSDMD) is a crucial effector molecule in the downstream inflammasome activation pathway and serves as a key executor protein in mediating pyroptosis [52]. Upon activation by the inflammasome, CASP1 cleaves GSDMD, resulting in the dissociation of its N-terminal domain (GSDMD-N). The dissociated GSDMD-N oligomerizes on the cell membrane to form pores, ultimately resulting in pyroptosis and the release of IL-1β and IL-18. Guo demonstrated that pyroptosis predominantly occurs during the initiation and maintenance phases of liver granulomatous inflammation following Schistosoma japonicum infection in mice [53]. Soluble egg antigen (SEA) alone can induce the activation of Caspase-1/4/11 in macrophages, subsequently leading to pyroptosis. We found that rEGR-01664 significantly increased the levels of CASP4 and GSDMD, but inhibited the production of IL-18 and IL-1β. Since the release of these cytokines depends on the formation of GSDMD pores, we speculate that rEGR-01664 may inhibit pyroptosis by blocking a key upstream event, such as the activation of inflammasomes or the proteolytic cleavage of GSDMD itself.

### 4.6. From Host Transmission to Human Disease: EGR-01664 as a Key Virulence Factor in E. granulosus Infection

This study reveals that EGR-01664 is a key effector molecule mediating the colonization and immune evasion of *E. granulosus* in both definitive and intermediate hosts. In the definitive host (dogs), the protein promotes the development of adult worms and the dissemination of eggs by shaping an immunosuppressive intestinal microenvironment, specifically by enhancing PBMCs proliferation, inhibiting NO production, and inducing an anti-inflammatory state (elevated IL-10, reduced IFN-γ). In human intermediate hosts, it drives the initiation and progression of CE through two mechanisms: first, inducing a Th2-biased immune response (suppression of pro-inflammatory cytokines and upregulation of IL-10); second, exerting dual regulation of cell death pathways—promoting apoptosis while inhibiting the release of pyroptosis-related inflammatory factors—thereby enabling the “silent” elimination of immune cells and ensuring the long-term survival of larval cysts.

## 5. Conclusions

The MEAs (EGR-01664) of *E. granulosus* exhibited a stable protein structure and prominent antigenic epitopes. Its prokaryotic expression product showed good immunogenicity. It is worth noting that, compared to the expression level of the protein in the adult stage, it was significantly upregulated in the protoscoleces stage, suggesting that the molecule may play a key role in the development and maturation of the parasites. In addition, the specific distribution characteristics of the recombinant protein EGR-01664 in the worms suggest that it may be involved in the interaction between *E. granulosus* and the host. More importantly, this protein can promote host cell apoptosis while inhibiting pyroptosis, indicating its potential mechanism for promoting immune escape by regulating the host immune response. This finding not only deepens our understanding of the pathogenic mechanism of *E. granulosus* but also provides new, important molecular targets for vaccine design and targeted therapy strategies in echinococcosis. Due to the scope of the current study, the immunogenicity of this antigen in definitive hosts has not yet been verified. Subsequent studies will systematically evaluate its immunoprotective efficacy and further elucidate its immunoregulatory mechanisms, aiming to provide experimental evidence for clinical applications.

## Figures and Tables

**Table 1:** The primer sequence information.

Gene	Primer Sequences (5′ → 3′)	Tm/°C	Product Length/bp	Applications
*EGR-01664*	GGAATTCGTCGCTGGAAAAGGCTACA CCAAGCTTTGACAATGCAGAACCCTCC	60.0	984	Cloning
*qEGR-01664*	AGAGGAGCCCAAGAATCCCT TGATCCAACTGGCACGTCAA	60.0	78	Real-time PCR
*β-actin*	AGTTCCTATGGTGTGCCTGC TCCCTCTTGGTCCGTGATCT	60.0	102	Real-time PCR
*TGF-β1*	TGCAAGTAGACATTAACGGGTTC GAAGGGTCGGTTCATGCCA	60.0	77	Real-time PCR
*IL-10*	CGCATTTAGTAAGCTCCAGG TGCCATCCTGGGTGTTTTGT	60.0	140	Real-time PCR
*IL-17A*	TTGGAATCTGCACCGCAATG TGGATGGGGACGGAGTTCAT	60.0	129	Real-time PCR
*IFN-γ*	CCAGATGTATCGGACGGTGG TGTTTTGTCACTCTCCTCTCTCC	60.0	72	Real-time PCR
*GSDMD*	GGCCTCCACACAGGTTTTCT ACTGAAGCGAGTCGTATGCC	60.0	72	Real-time PCR
*Bcl-2*	GACTGAGTACCTGAACCGGC AGTTCCACAAAGGCATCCCAG	60.0	72	Real-time PCR
*Bax*	GCCCTTTTGCTTCAGGGTTTC CGATGCGCTTGAGACATTCG	60.0	130	Real-time PCR
*GASP4*	CAGGCCTGCAGAGGTGAAAA GGCATCACTCTGGAGCATCA	60.0	105	Real-time PCR
*IL-18*	TGAACGACCAAGTCCTCTTCG TGCCAGACCTCTAGTGAGGC	60.0	140	Real-time PCR
*IL-1β*	CCTGGAAATGTGAAGTGCTGC TTGCAACTGGATGCCCTCAT	60.0	72	Real-time PCR

**Table 2:** Bioinformatic analysis of EGR-01664.

Gene	Amino Acid (aa)	Molecular Weight (kDa)	PI	Instability Index	Signal Peptide	Transmembrane Area	B-Cell Antigenic Epitope (Score > 0.85)
*EGR-01664*	343	38.09	6.88	58.41	—	—	8

## Data Availability

All data are contained within the manuscript. For further details, please contact the corresponding author.

## References

[1] Yang Z., Liu K., Wen B., Fu T., Qin X., Li R., Lu M., Wang Y., Zhang W., Shao Z. (2024). Changes in the global epidemiological characteristics of cystic echinococcosis over the past 30 years and projections for the next decade: Findings from the Global Burden of Disease Study 2019. J. Glob. Health.

[2] Van De N., Le Van D. (2017). The first report of two cases of cystic echinococcosis in the lung by Echinococcus ortleppi infection, in Vietnam. Res. Rep. Trop. Med..

[3] Xu X., Qian X., Gao C., Pang Y., Zhou H., Zhu L., Wang Z., Pang M., Wu D., Yu W. (2022). Advances in the pharmacological treatment of hepatic alveolar echinococcosis: From laboratory to clinic. Front. Microbiol..

[4] Deplazes P., Rinaldi L., Alvarez Rojas C.A., Torgerson P.R., Harandi M.F., Romig T., Antolova D., Schurer J.M., Lahmar S., Cringoli G. (2017). Global Distribution of Alveolar and Cystic Echinococcosis. Adv. Parasitol..

[5] Zhu G.Q., Yan H.B., Li L., Jia W.Z. (2019). Research progress on hydatid disease vaccines. Chin. J. Zoonoses.

[6] Zhou X.H., Wu J.Y., Huang X.Q., Kunnon S.P., Zhu X.Q., Chen X.G. (2010). Identification and characterization of Schistosoma japonicum Sjp40, a potential antigen candidate for the early diagnosis of schistosomiasis. Diagn. Microbiol. Infect. Dis..

[7] Rostami-Rad S., Jafari R., Yousofi Darani H. (2018). Th1/Th2-type cytokine profile in C57 black mice inoculated with live Echinococcus granulosus protoscolices. J. Infect. Public Health.

[8] Biosa G., Bonelli P., Pisanu S., Ghisaura S., Santucciu C., Peruzzu A., Garippa G., Uzzau S., Masala G., Pagnozzi D. (2022). Proteomic characterization of Echinococcus granulosus sensu stricto, Taenia hydatigena and Taenia multiceps metacestode cyst fluids. Acta Trop..

[9] Li Q., Zhang J., Fan B., Wang F. (2022). Bioinformatics analysis of the molecular characteristics of the main egg antigen proteins of Echinococcus. Chin. Trop. Med..

[10] Wang J., Gottstein B. (2016). Immunoregulation in larval Echinococcus multilocularis infection. Parasite Immunol..

[11] Cao S., Gong W., Zhang X., Xu M., Wang Y., Xu Y., Cao J., Shen Y., Chen J. (2020). Arginase promotes immune evasion of Echinococcus granulosus in mice. Parasites Vectors.

[12] Amri M., Mezioug D., Touil-Boukoffa C. (2009). Involvement of IL-10 and IL-4 in evasion strategies of Echinococcus granulosus to host immune response. Eur. Cytokine Netw..

[13] Shao S., Sun X., Chen Y., Zhan B., Zhu X. (2019). Complement Evasion: An Effective Strategy That Parasites Utilize to Survive in the Host. Front. Microbiol..

[14] Folle A.M., Lagos Magallanes S., Fló M., Alvez-Rosado R., Carrión F., Vallejo C., Watson D., Julve J., González-Sapienza G., Pristch O. (2024). Modulatory actions of Echinococcus granulosus antigen B on macrophage inflammatory activation. Front. Cell. Infect. Microbiol..

[15] Mamuti W., Sako Y., Nakao M., Xiao N., Nakaya K., Ishikawa Y., Yamasaki H., Lightowlers M.W., Ito A. (2006). Recent advances in characterization of Echinococcus antigen B. Parasitol. Int..

[16] Siracusano A., Riganò R., Ortona E., Profumo E., Margutti P., Buttari B., Delunardo F., Teggi A. (2008). Immunomodulatory mechanisms during Echinococcus granulosus infection. Exp. Parasitol..

[17] Du X. (2022). Molecular Identification of Echinococcus Granulosus Ubiquitin-Conjugating Enzymes E2D2 and E2N and Their Effects on the Formation of Host Liver Fibrosis.

[18] Wang Y., Guo A., Zou Y., Mu W., Zhang S., Shi Z., Liu Z., Cai X., Zhu X.Q., Wang S. (2023). Interaction between tissue-dwelling helminth and the gut microbiota drives mucosal immunoregulation. NPJ Biofilms Microbiomes.

[19] Wang Z., Pu N., Zhao W., Chen X., Zhang Y., Sun Y., Bo X. (2024). RNA sequencing reveals dynamic expression of genes related to innate immune responses in canine small intestinal epithelial cells induced by Echinococcus granulosus protoscoleces. Front. Vet. Sci..

[20] Urban B.C., Ferguson D.J., Pain A., Willcox N., Plebanski M., Austyn J.M., Roberts D.J. (1999). Plasmodium falciparum-infected erythrocytes modulate the maturation of dendritic cells. Nature.

[21] Wei X.L., Xu Q., Rexiti F.L., Zhu M., Lin R.Y., Wen H. (2014). Dynamic changes of DC and T cell subsets in mice during Echinococcus multilocularis infection. Cent. -Eur. J. Immunol..

[22] Wang J., Müller S., Lin R., Siffert M., Vuitton D.A., Wen H., Gottstein B. (2017). Depletion of FoxP3+ Tregs improves control of larval Echinococcus multilocularis infection by promoting co-stimulation and Th1/17 immunity. Immun. Inflamm. Dis..

[23] Xian J., Wang N., Zhao P., Zhang Y., Meng J., Ma X., Wang Z., Bo X. (2021). Molecular Characterization and Expression Analysis of the Gene Encoding 3-Hydroxyacyl-CoA Dehydrogenase (EGR-03347) from Echinococcus Granulosus and the Evaluation of the Immune Protection of the Definitive Hosts (dogs). Parasites Vectors.

[24] Shao G., Zhu X., Hua R., Lu Z., Chen Y., Yang A., Yang G. (2025). Cocktail vaccine induces immunoprotection and modulates the fecal microbiota in dogs against Echinococcus granulosus infection. NPJ Vaccines.

[25] Wen H., Vuitton L., Tuxun T., Li J., Vuitton D.A., Zhang W., McManus D.P. (2019). Echinococcosis: Advances in the 21st Century. Clin. Microbiol. Rev..

[26] Jiang J., Li J., Zhang Y., Zhou C., Guo C., Zhou Z., Ming Y. (2022). The Protective Effect of the Soluble Egg Antigen of Schistosoma japonicum in A Mouse Skin Transplantation Model. Front. Immunol..

[27] Abouel-Nour M.F., Lotfy M., Attallah A.M., Doughty B.L. (2006). Schistosoma mansoni major egg antigen Smp40: Molecular modeling and potential immunoreactivity for anti-pathology vaccine development. Mem. Do Inst. Oswaldo Cruz.

[28] Zhang J.M., Li R., Chen X.Q., He X.D., Wang Z.R., Zheng Y.D., Guo X.L. (2021). Preparation and preliminary application of recombinant major egg antigen protein of *Echinococcus multilocularis* in immunodiagnosis. Chin. Vet. Sci..

[29] La Mendola D., Magrì A., Campagna T., Campitiello M.A., Raiola L., Isernia C., Hansson O., Bonomo R.P., Rizzarelli E. (2010). A doppel alpha-helix peptide fragment mimics the copper (II) interactions with the whole protein. Chemistry.

[30] Marcos E., Chidyausiku T.M., McShan A.C., Evangelidis T., Nerli S., Carter L., Nivón L.G., Davis A., Oberdorfer G., Tripsianes K. (2018). De novo design of a non-local β-sheet protein with high stability and accuracy. Nat. Struct. Mol. Biol..

[31] Lin W., Liang W.C., Nguy T., Maia M., Tyagi T., Chiu C., Hoi K.H., Chen Y., Wu Y. (2020). Rapid identification of anti-idiotypic mAbs with high affinity and diverse epitopes by rabbit single B-cell sorting-culture and cloning technology. PLoS ONE.

[32] Wu L.Y., Wang Y.J., Wen Y.L., Yan R.F., Xu L.X., Song X.K., Li X.R. (2017). Cloning, expression and functional analysis of NADH: Ubiquinone oxidoreductase domain-containing protein gene from *Haemonchus contortus*. Acta Vet. Zootech. Sin..

[33] Wang B., Zhang D.M., Pan W.Q. (2006). Detection of lymphocyte proliferation stimulated by plasmodium HGXPRT protein using CCK-8 assay. China Trop. Med..

[34] Wu X., Sun L., Zhang L., Liu Z.Q., Luo Q., Zhang L.X. (2012). Effects of *Toxoplasma gondii* on proliferation and apoptosis of four types of tumor cells. Chin. J. Parasitol. Parasit. Dis..

[35] Yang X., Chen T., Yu X. (2016). The research progress of inducible nitric oxide synthase and parasitic infection. Chin. J. Pathog. Biol..

[36] Bogdan C. (2015). Nitric oxide synthase in innate and adaptive immunity: An update. Trends Immunol..

[37] Ehsan M., Gao W., Gadahi J.A., Lu M., Liu X., Wang Y., Yan R., Xu L., Song X., Li X. (2017). Arginine kinase from Haemonchus contortus decreased the proliferation and increased the apoptosis of goat PBMCs in vitro. Parasites Vectors.

[38] Raphael I., Nalawade S., Eagar T.N., Forsthuber T.G. (2015). T cell subsets and their signature cytokines in autoimmune and inflammatory diseases. Cytokine.

[39] Buckner J.H. (2010). Mechanisms of impaired regulation by CD4 (+) CD25 (+) FOXP3 (+) regulatory T cells in human autoimmune diseases. Nat. Rev. Immunol..

[40] Shevach E.M. (2002). CD4+ CD25+ suppressor T cells: More questions than answers. Nat. Rev. Immunol..

[41] Frank D., Vince J.E. (2019). Pyroptosis versus necroptosis: Similarities, differences, and crosstalk. Cell Death Differ..

[42] Chen Y., Hua R., Shao G., Zhu X., Hou W., Li S., Yang A., Yang G. (2024). Effects of annexin B18 from Echinococcus granulosus sensu lato on mouse macrophages. Exp. Parasitol..

[43] He X., Shao G., Du X., Hua R., Song H., Chen Y., Zhu X., Yang G. (2023). Molecular characterization and functional implications on mouse peripheral blood mononuclear cells of annexin proteins from Echinococcus granulosus sensu lato. Parasites Vectors.

[44] Kachler K., Holzinger C., Trufa D.I., Sirbu H., Finotto S. (2018). The role of Foxp3 and Tbet co-expressing Treg cells in lung carcinoma. Oncoimmunology.

[45] Salim T., Sershen C.L., May E. (2016). Investigating the Role of TNF-α and IFN-γ Activation on the Dynamics of iNOS Gene Expression in LPS Stimulated Macrophages. PLoS ONE.

[46] Zhao S., Zhang Y., Lu X., Ding H., Han B., Song X., Miao H., Cui X., Wei S., Liu W. (2021). CDC20 regulates the cell proliferation and radiosensitivity of P53 mutant HCC cells through the Bcl-2/Bax pathway. Int. J. Biol. Sci..

[47] Hao Q., Chen J., Lu H., Zhou X. (2023). The ARTS of p53-dependent mitochondrial apoptosis. J. Mol. Cell Biol..

[48] Chao R., Hu X.Y., Zhu S.D., Deng W., Wang L. (2019). Effects of matrine combined with chemotherapeutic drugs on proliferation and invasiveness of human acute myeloid leukemia HL-60 cells. World J. Tradit. Chin. Med..

[49] Liu Z., Wang C., Yang J., Chen Y., Zhou B., Abbott D.W., Xiao T.S. (2020). Caspase-1 Engages Full-Length Gasdermin D through Two Distinct Interfaces That Mediate Caspase Recruitment and Substrate Cleavage. Immunity.

[50] Aglietti R.A., Estevez A., Gupta A., Ramirez M.G., Liu P.S., Kayagaki N., Ciferri C., Dixit V.M., Dueber E.C. (2016). GsdmD p30 elicited by caspase-11 during pyroptosis forms pores in membranes. Proc. Natl. Acad. Sci. USA.

[51] Devant P., Dong Y., Mintseris J., Ma W., Gygi S.P., Wu H., Kagan J.C. (2023). Structural insights into cytokine cleavage by inflammatory caspase-4. Nature.

[52] Wei C., Jiang W., Wang R., Zhong H., He H., Gao X., Zhong S., Yu F., Guo Q., Zhang L. (2024). Brain endothelial GSDMD activation mediates inflammatory BBB breakdown. Nature.

[53] Yin G., Qi X., Li Y., Xu L., Zhou S., Chen X., Zhu J., Su C. (2022). The mechanism of mouse macrophage apoptosis induced by *Schistosoma japonicum* soluble egg antigen. Chin. J. Schistosomiasis Control.

